# Features of the Correlation Structure of Price Indices

**DOI:** 10.1371/journal.pone.0061091

**Published:** 2013-04-08

**Authors:** Xiangyun Gao, Haizhong An, Weiqiong Zhong

**Affiliations:** 1 School of Humanities and Economic Management, China University of Geosciences, Beijing, China; 2 Key Laboratory of Carrying Capacity Assessment for Resource and Environment, Ministry of Land and Resource, Beijing, China; 3 Lab of Resources and Environmental Management, China University of Geosciences, Beijing, China; Umeå University, Sweden

## Abstract

What are the features of the correlation structure of price indices? To answer this question, 5 types of price indices, including 195 specific price indices from 2003 to 2011, were selected as sample data. To build a weighted network of price indices each price index is represented by a vertex, and a positive correlation between two price indices is represented by an edge. We studied the features of the weighted network structure by applying economic theory to the analysis of complex network parameters. We found that the frequency of the price indices follows a normal distribution by counting the weighted degrees of the nodes, and we identified the price indices which have an important impact on the network's structure. We found out small groups in the weighted network by the methods of k-core and *k*-plex. We discovered structure holes in the network by calculating the hierarchy of the nodes. Finally, we found that the price indices weighted network has a small-world effect by calculating the shortest path. These results provide a scientific basis for macroeconomic control policies.

## Introduction

Price indices are important indicators for measuring inflation and economic development. The government influences commodity prices by a variety of regulatory policies to maintain the stability of the market. Price indices include the residents' consumer price index (CPI), the producer price index (PPI), the retail price index (RPI), the agricultural production and agricultural production material price index (API), and the raw materials, fuel and power purchase prices index (RFPPI) [1]–[9]. These 5 types of price indices are divided into many specific price indices. For example, in China, the CPI is classified into eight categories: food, tobacco and liquor, clothing, household equipment and services, health care and personal products, transport and communications, entertainment and educational products, and services and housing. These 5 types of price indices are obtained by calculating specific price indices with weights. Thus, compared with CPI, PPI, RPI, API, and RFPPI, specific price indices more directly reflect changes in commodity prices. For this reason, we study specific price indices instead of these 5 types of price indices.

There are relationships between price indices that have been found in previous research. Models and the methods of econometrics are applied to prove that there are causal relationships [4], cointegration relationships [5], interactive relationships [10], and transmission relationships [11]–[13] among the 5 price indices. These relationships are based on the correlation between price indices [14]. However, there are many specific price indices for each type, among which the perplexing relationships form a complicated network [15]–[17]. In this price indices network, which price indices have a greater impact? How do they affect each other? When one of them changes, which price indices will transmit fluctuation impact? How far can the transmission process go, and what is the transmission path? Because traditional econometric models cannot include so many price indices, if we do so, the model will be too difficult to use [14]. To answer the 4 questions above, we should first find out the features of the relationship structure between the price indices. Complex network theory can effectually solve problems regarding the features of the relationship structure. The core concept of complex network theory is to view the relationship between the variables in the real system as a complex network, to describe the relationship between the variables in the real complex system in the form of a network, and to obtain a better understanding of its nature by analyzing the structure of the system [18]–[20]. This theory provides an adequate approach to quantitatively analyze the behavior of complex networks in the economic system, such as small-world phenomenon [16], [21]. The diversity of individuals' relationships is strongly correlated with the economic development of communities [22]. Researchers have analyzed the individuals' relationships between stock market indices based on correlation network. They found out the features of volatility and the dominant stocks in stock market indices [23], [24], [25]. In economic system, hundreds of price index interact and affect each other, and the correlation structure forming a complex network. Thus, we can analyze the correlation structure by complex network theory.

Therefore, this paper aims to provide a method for the study of multivariate relationships in hundreds of variables by using complex network theory. We will answer the 4 questions above by defining the relationships between the price indices, building up a price index weighted network and studying the features of the correlation structure that are based on complex network theory.

## Materials and Methods

### Materials

This study collected data on specific price indices from 2003–2011 of the developing country China (China Statistical Yearbook, 2003–2011). Data from the following five types of price indices were used: CPI, RPI, API, PPI, and RFPPI. Each price index is divided into specific price indices, with a total of 195 indices. The main elements of the various types of price indices are shown in Table 1, for example, the CPI food index includes a food price index, an oil price index, a meat and poultry price index, a water product price index, and other food price indices.

**Table 1:** Major elements of price indices.

Type of price index	Main elements
Consumer Price Index(CPI)	Food, Tobacco, Liquor and Articles, Clothing, Household Facilities, Articles and Services, Health Care and Personal Articles, Transportation and Communication, Recreation, Education and Culture Articles, Residence, et al.
Retail Price Index(RPI)	Food, Beverages, Tobacco and Liquor, Garments, Shoes and Hats, Textiles, Household Appliances, Music and Video Equipment, Cultural and Office Appliances, Articles for Daily Use, Sports and Recreation Articles, Transportation and Communication Appliances, Furniture, Cosmetics, Gold, Silver and Jewelry, Traditional Chinese and Western Medicines and Health Care Articles, Books, Newspapers, Magazines and Electronic Publications, Fuels, Building Materials and Hardware, et al.
Producer Price Index for Manufactured Goods(PPI)	Metallurgical Industry, Power Industry, Coal Industry, Petroleum Industry, Household Appliances, Music and Video Equipment, Machine Manufac-turing Industry, Building Materials Industry, Timber Industry, Food Industry, Textile Industry, Tailoring Industry, Leather Industry, Paper Industry, Cultural, Educational and Handicrafts Articles, et al.
Agricultural Products Price Index and Price Index of Agricultural Pruductive Material(API)	Planting Products, Forestry Products, Animal Husbandry Products, Fishery Products, Household Appliances, Music and Video Equipment, Forage, Commodity Animals, Semi-mechanized Farm Tools, Mechanized Farm Machinery, Chemical Fertilizer, Pesticide and Its Appliances, Oil for Farm Machinery, Other Means of Agricultural Production, Service for Agricultural Production, et al.
Purchasing Price Index for Raw Material, Fuel and Power(RFPPI)	Fuel and Power, Ferrous Metals, Nonferrous Metals, Raw Chemical Materials, Household Appliances, Music and Video Equipment, Building Materials, Agricultural Products, Textile Materials, et al.

### Price indices weighted network

To study the features of the correlation structure of specific price indices, we first should establish the price index weighted network (PIWN) by complex network theory. A network is a collection of vertices and edges, *N* = (*V, E*). We represented every price index as a vertex, and we represented the relationship between two price indices as an edge in the network; thus, we turned the study of the correlation structure of price indices into a study of a complex network of price correlations.

The set of vertices *V* in the complex network of price index correlations is expressed as
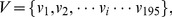
(1)where *v_i_* represents the *i*-th price index.

The set of edges *E* in the complex network of price index correlations is expressed as
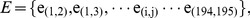
(2)where *e_(i, j)_* represents the relationship between the *i*-th price index and the *j*-th price index.

In the course of this study, we aimed to define and quantify the network's relevance. We quantify the edge *e_(i, j)_* and use the correlation coefficient *r_ij_* to represent the correlation degree between the *i*-th price index and the *j*-th price index. The correlation coefficient, also known as the Pearson correlation coefficient, is an indicator that measures the degree of correlation between changing trends of variables, with a range of [−1,1]. The greater the absolute value of the correlation coefficient, the higher the degree of correlation between the variables.
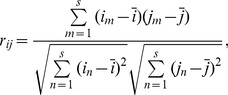
(3)where *i_m_* is the time series value of the *i*-th price index, 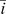 is the average time series value of the *i*-th price index, *j_n_* is the time series value of the *j*-th price index, 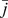 is the average time series value of the *j*-th price index, and *s* is the number of items in the price index series.

Thus, the price index correlation coefficient matrix *R* is formed as
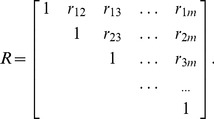
(4)


If all of the correlation coefficients are reflected in the network with weights, then the network is completely connected and it becomes unavailable for topology analysis. Furthermore, to draw the relationship and structure between the price indices more accurately, we should remove the weak correlations and non-correlations by setting a threshold. The PIWN is a threshold-based network, and like all threshold-based networks, it is very sensitive to the value of the threshold. Edges whose weights are less than the threshold value can be omitted [23], [24]. As the threshold increases, the network becomes more informative about the partial correlation structure of the system, but partial correlation selection could be affected by statistical uncertainty. The threshold value is not supposed to be too low. In economic networks, the threshold is usually set to be above 0.7 [23], [25].

**Figure 1:**
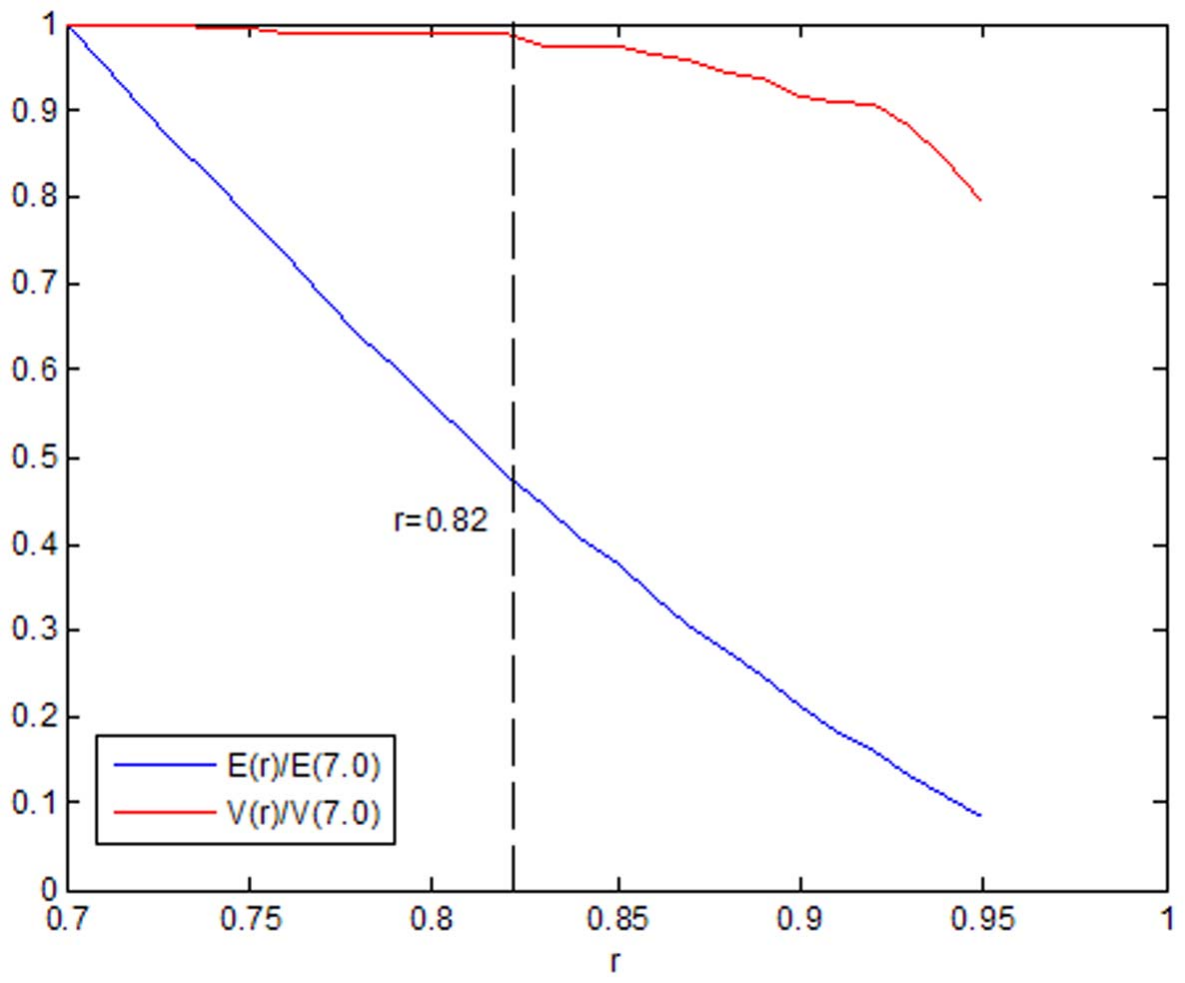
Two measures of PIWN connectivity as a function of the parameter *r*. The value *r* = 0.82 is the measure used in this paper.

**Figure 2:**
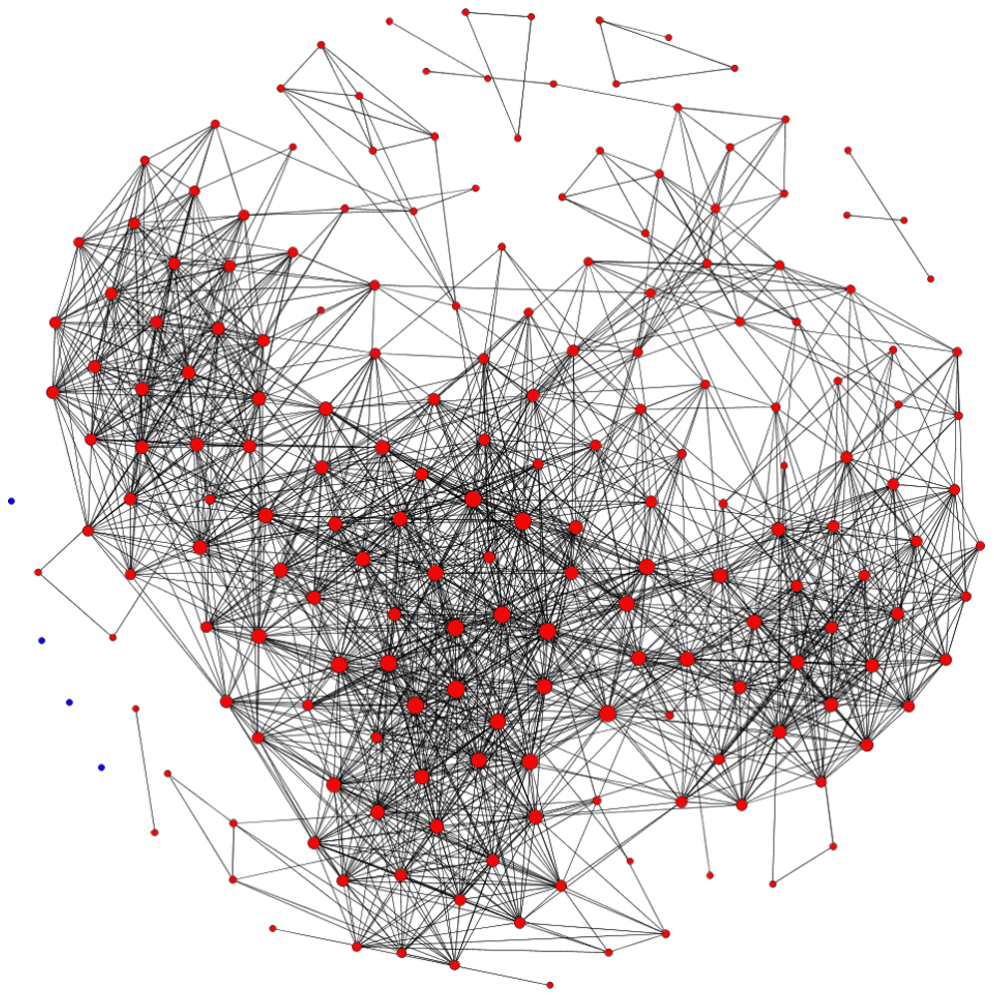
Complex network of strength price index correlations. The 4 blue nodes are isolated nodes that have no strong correlation with any other nodes.

The topological and metric properties of the PIWN depend strongly on the value of the correlation coefficient *r*. To select a suitable value for *r*, we iteratively choose different values of this correlation coefficient and compute the sum of the weights of all of the edges in the resulting PIWN. We represent this quantity as *E (r)*. For *r* = 0.7, we have *E (0.7)* = 3734. We report the fraction 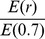 as a function of r. In addition, we perform a similar analysis for the size of the largest connected component in the network, depending on the value of *r*. We indicate the total number of vertices in the largest connected component of the PIWN for a given *r* with *V(r)*. In figure 1, we show the quantity 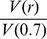, where *r* = 0.82 is the break point. Thus, we choose 0.82 as our threshold value for the size of a large connected component and non-trivial topological and metric properties of the resulting price index network [24]. If the correlation coefficient between two price indices is not less than 0.82, then there is an edge between them. The value of the correlation coefficient between two price indices is set to be the weight of the edge. Thus, we build up a weighted complex network structure model of strong correlations of price indices, as shown in Figure 2.

## Results

The 4 questions on the PIWN mentioned above are managed by the method of complex network parametric analysis, including the weighted degree, *k*-core, *k*-plex, the hierarchy of structural holes and the shortest path.

### Key price indices

Which price indices have a larger impact? To answer this question, we must find the key price indices in the PIWN. Key price indices are the indicators that have a very high impact in the PIWN; the higher the weighted degree of a node is, the wider its impact can reach. In economic systems, the wider the impact of an index can reach, the more important it is in the network. Thus, we can find key price indices in the PIWN by calculating the weighted degree of the nodes. The weighted degree of a node is the sum of all of the weighted values of its edges. This sum not only includes the number of price indices related to the node but also considers the degree of their correlations. The weighted degree *WD_i_* of a price index (vertex) *i* is defined as
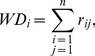
(5)where *r* is the value of the correlation coefficient between the vertices *i* and the vertices *j*. In Figure 2, the larger the value is, the larger the node is in the network.

**Figure 3:**
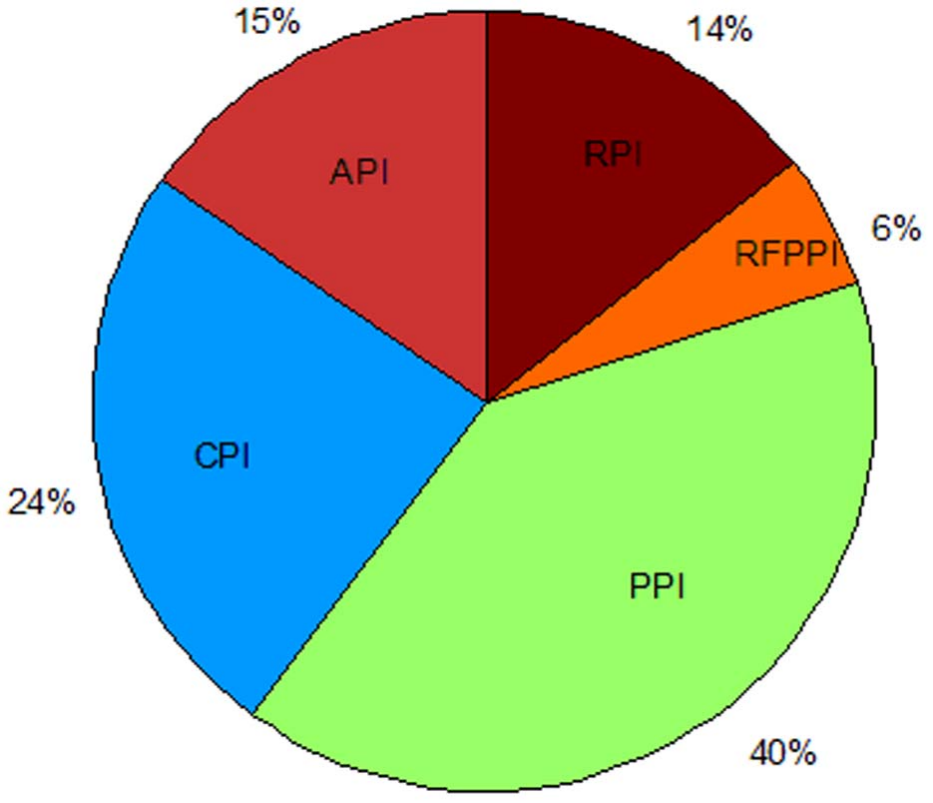
Proportions of macro price indices with correlation relationships.

Our experiment includes 191 vertices and 1,795 edges in the PIWN. We calculated the weighted degree of the 191 nodes, reflected it to the macro-level, and found that, in the whole price index correlation network structure, the degree of influence of the PPI is the widest. Thus on the macro-level, PPI is the key price index. The PPI refers to the producer price index in China. In studies on the price transmission relationship in the industrial chain, many scholars believe that the PPI can be used to represent the upstream price level in the industry transmission chain. Second, the degree of influence of the CPI is 24%, and this index can be used in lieu of the downstream price in the industry transmission chain. The results show that the PPI has a larger impact on the inflation rate than the CPI. The results of other studies also support this conclusion. Using empirical analysis, the transmission mechanism from the production price to the consumer price is more important than the transmission mechanism from the consumer price to the production price [11]. In general, fluctuations in the overall price level appear first in the production area and, from there, spread through the industrial chains to the downstream industries and, finally, to consumer goods [12]. Thus, to control the inflation rate in China, the upstream of the industrial chain can be considered first. The results also show that the degree of influence of the API is up to 15%. China is an agricultural country; thus, the impact of the price level of agricultural production and its impact on other industries should not be underestimated. Especially in developing countries, an increase in people's basic costs of living often has a series of consequences, such as the recent “pork inflation” in China. Three types of price indices, the PPI, the CPI, and the API, cover 80% of the degree of influence. The statistics of the correlation between the macro price indices are shown in Table 2, and the proportion of every index is shown in Figure 3.

**Table 2:** Weighted degree of the macro price index.

Type of price index	Weighted Degree
PPI	1286.29
CPI	787.21
API	496.98
RPI	463.55
RFPPI	182.89

In Table S1, we provide all values of the weighted degree of the price index. There are only a small number of key price indices in the PIWN. Only 3 key price indices have weighted degrees that are above 40. The value of the weighted degree of the price index (top 10) is shown in Table 3. This table shows that, in the whole price indices system, only a few price indices have impacts that are larger than 40. Changes in these key price indices will lead to fluctuations in the other price indices and will lead to some economic phenomenon of the entire system. For example, China is the world's most populous country. The cost of living of the people occupies a pivotal position in its economic development; as a result, fluctuations in the daily life and the retail, consumer price indices are often the cause of inflation [26].

**Table 3:** The value of the weighted degree of the price index (top 10).

Rank	Type of price index	Weighted degree
1	RPI(Rural Household)	42.19
2	PPI(Articles for Daily Use)	41.70
3	RPI	40.59
4	PPI(Manufacture of General Purpose Machinery)	39.92
5	RPI(Urban Household)	39.64
6	CPI(Rural Household)	39.14
7	PPI(Manufacture of Special Purpose Machinery)	39.00
8	PPI(Manufacture of Artwork and Other Manufacturing)	38.63
9	RPI(Building Materials and Hardware)	38.58
10	PPI(Consumer Goods)	37.62

**Figure 4:**
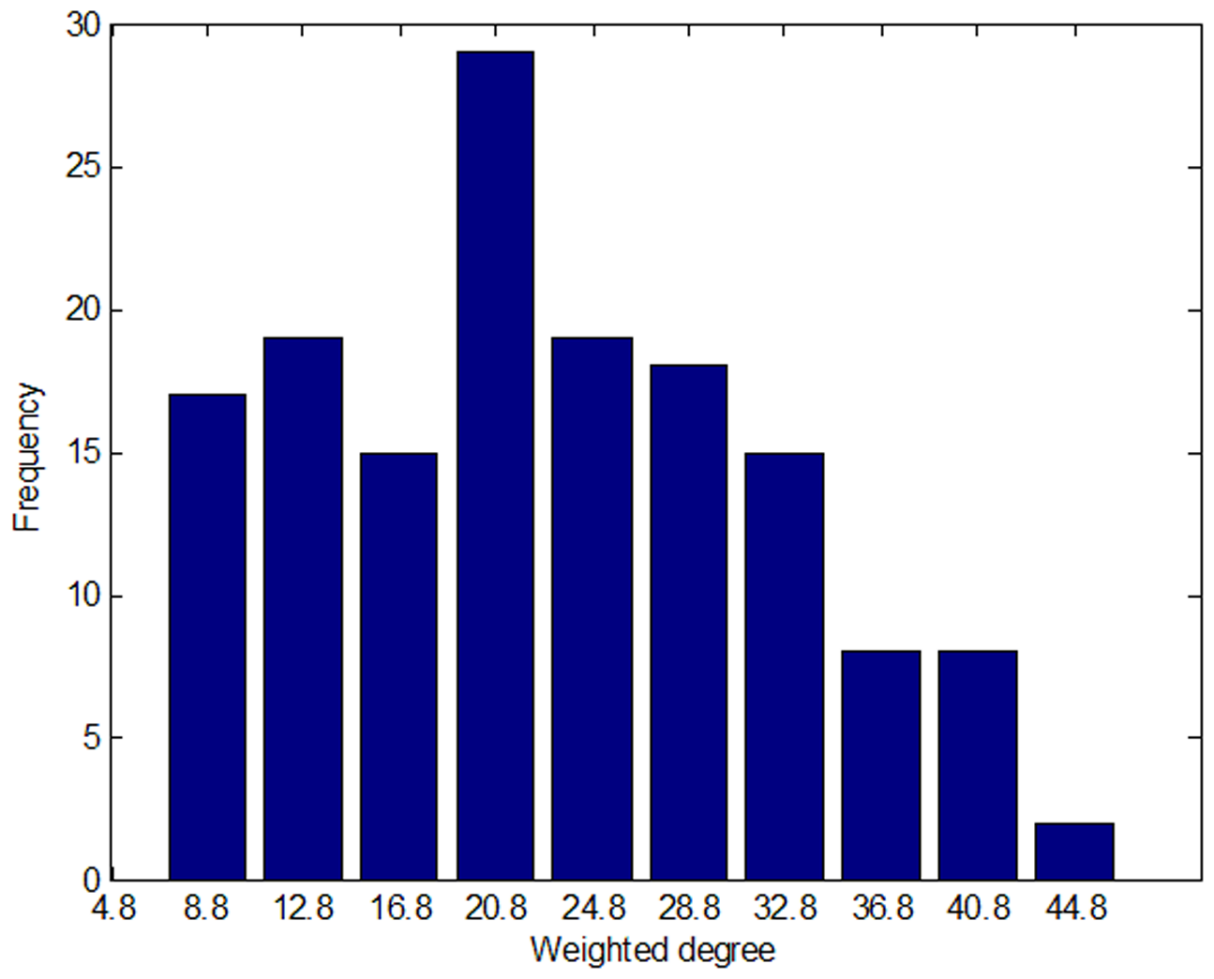
Distribution of the price index. A weighted degree between 4.8 and 44.8, following a normal distribution with a confidence level of 0.77.

We found that there are as many as 41 weighted degrees whose value is under 4.8. In figure 2, the nodes in the sparse part of the network structure are small, which means that they carry less impact compared with other price indices and stay in relatively independent states. By analyzing price indices with weighted degrees between 4.8 and 44.8, we found that they follow a normal distribution, as shown in Figure 4. The leading roles of the price index system are occupied by the middle indices, price indices with weighted degrees between 8.8 and 32.8. These results can be used as a reference to decide on key directions for inflation regulation in China. However, in the overall PIWN, there are some relatively independent group clusters and a transmission medium between the price indices that must be studied further.

### Group clusters

How do the price indices affect each other? To answer this question, we should study the group clusters in the PIWN. Group clusters are sub-networks in which the price indices have strong correlations. In a real economic system, many commodity price indices interact with each other. Changes in one price index tend to drive changes in another price index; there is a mutually strong relationship between these price indices. As observed in Figure 2, the entire network includes some small network group clusters, which are in a relatively independent state. We discovered that the group clusters of price indices can help us to understand the features of the price indices' correlation structure and provide better references for controlling policies. The *k*-plex and the *k*-core method are based on the discovery of vertex degrees in subgroups; they are useful in identifying small groups of clusters in the network structure of the price index. Using the subgroup discovery method, the group clusters in the price index are tapped, which can help when attempting to understand which other indices change when a specific price index changes.

The *k*-plex method requires that each vertex of the *g* vertices that are included in a subgroup maintains at least *g*-*k* links with other vertices in the same subgroup, where *k* is an adjustment coefficient; the smaller the value of *k* is, the larger the value of *g*. Furthermore, the more demanding conditions there are, the closer the relationship between the vertices. The *k*-core method refers to a subgraph with the following conditions: the points in the subgraph are at least adjacent to *k* other points in the subgraph. The *k*-plex method requires that, in addition to the *k* point, the points be connected with at least one point outside the *k* points, whereas the *k*-core method requires every point to be connected to at least *k* points.

**Figure 5:**
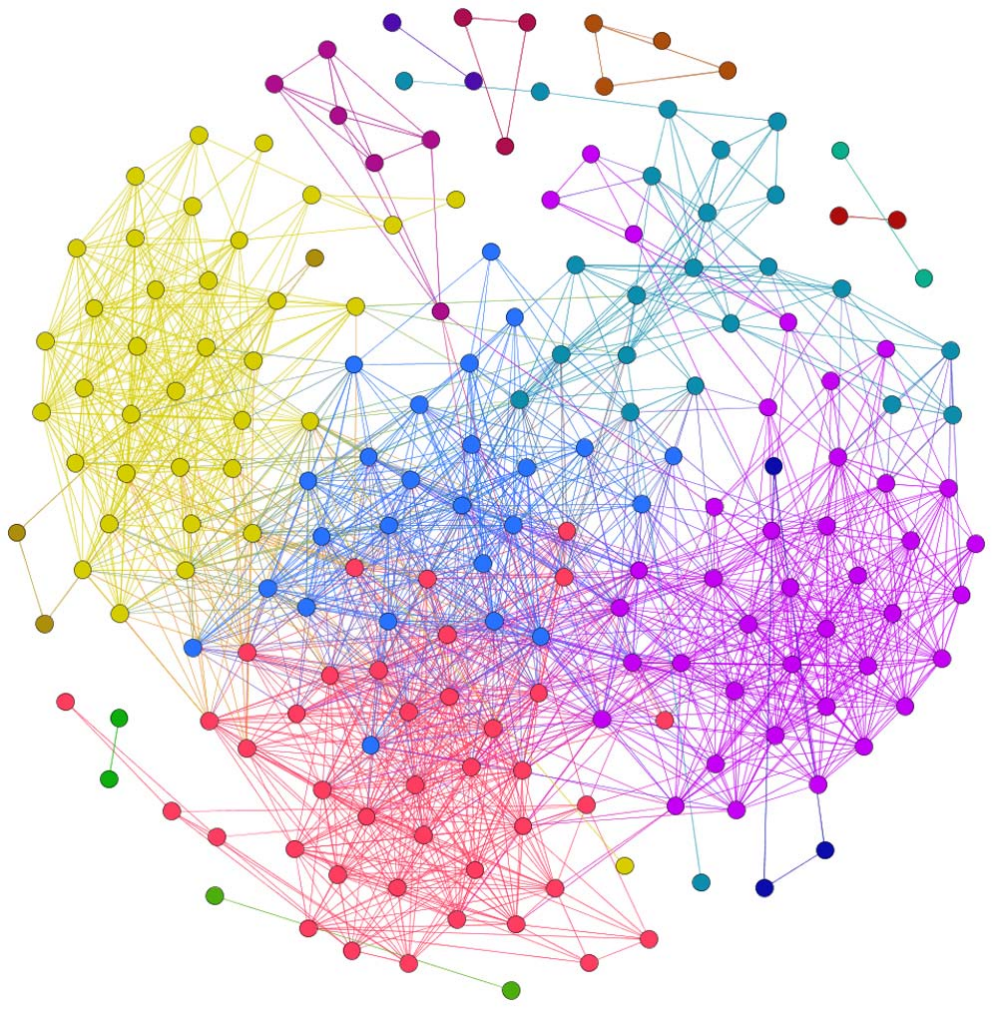
The *k*-core in the Price index correlation network structure. The class of group cluster is shown in Table S2.

**Figure 6:**
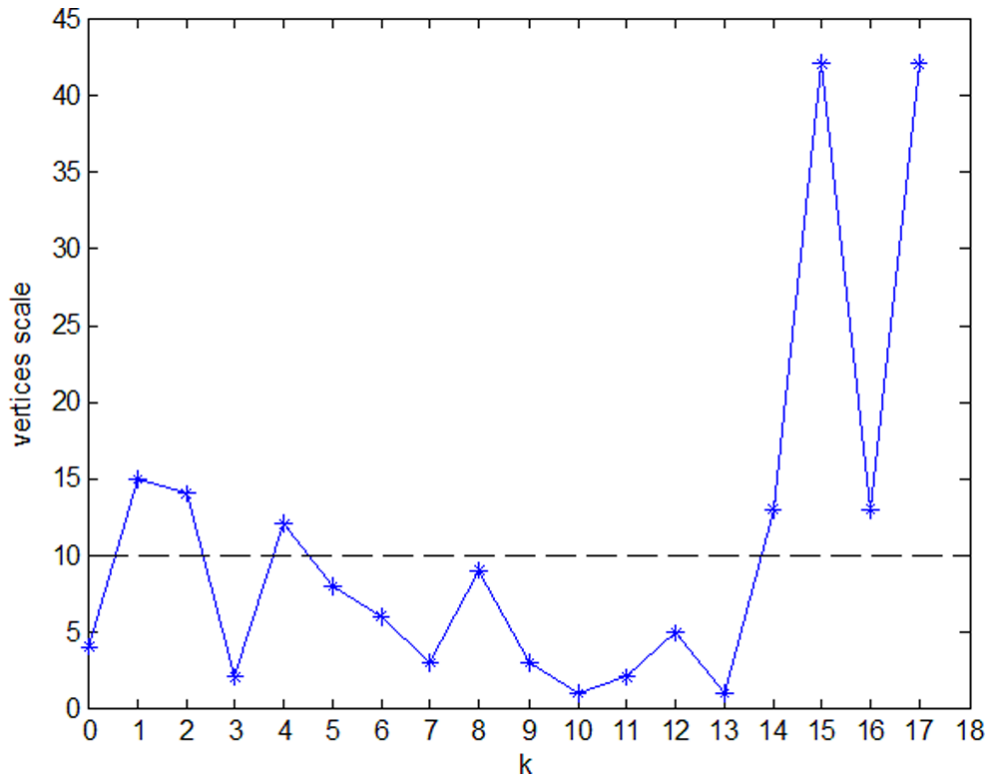
The *k*-core vertices scale in the price index correlation network structure.

First, use the *k*-core method to find *k*-core in the PIWN. The experimental results show that there are 18 group clusters (Figure 5 shows different colors representing different *k*-cores). There are seven groups with vertex scales of no less than 10 (the vertex scale represents the number of price indices in a group cluster), as shown in Figure 6. The largest group clusters contain 42 price indices. As shown in Figure 5, there are 3 large group clusters in the PIWN, including 43% of the price indices and 54% of the correlations. These 3 group clusters are in significant positions; any price index in the group cluster changes will arouse changes in the others and lead to changes in the whole. Thus, the government could control the fluctuation in the price indices by dispersed regulation to maintain the stability of the whole system.

**Figure 7:**
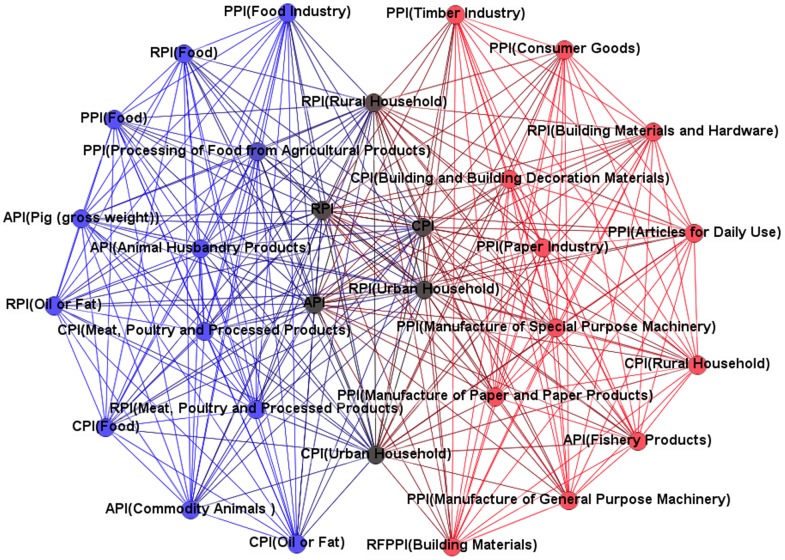
Group clusters. *g* = 17, *k* = 2.

Subsequently, the *k*-plex method is used to search for group clusters in the PIWN under the most demanding conditions by adjusting the value of *k* and the vertices scale. In the experiment, we set the vertices scale to *g* = 17 and the adjustment coefficient to *k* = 2, and only 2 group clusters are found. The 2 group clusters are price indices collections that have the tightest correlations in the whole network structure, as shown in Figure 7. Red vertices and blue vertices represent two groups of clusters, and the black vertices are the common price indices for these two group clusters. As observed in Figure 7, the common price indices are all macroeconomic price indices that are composed of the CPI, the RPI and the API. From constituent elements of the 2 group clusters, we can find that, in the PIWN, there are 2 group clusters with tight correlations; one cluster comprises food price indices and the other cluster comprises consumer goods price indices of the PPI. These two group clusters have strong positive relationships with macro price indices, which means that it is effective for China to regulate and stabilize price markets via the food and upstream price indices.

### Transmission medium

When one of the price indices changes, which price indices will transmit fluctuation impact? To answer this question, we must analyze the intermediary of every price index in the PIWN. In economic systems, the fluctuation of any commodity price can cause changes in other commodity prices, and this fluctuation is transitive. This transmission effect is based on correlations among the commodity prices. Similar to controlling the spread of a virus, we should not only control the source of the virus but also stop the transmission by eliminating the mass medium. In an economic system, the price index in the network structure acts not only as the source and receptor of the transmission process but also as the medium. Different price indices have different levels of medium effects. We could control the transmission of fluctuations between price indices by controlling the mediums, to avoid the influence of the whole system.

To seek for the price index that plays the role of the intermediary, we must analyze the hierarchy of structural holes for each price index in the PIWN. The presence of structural holes makes the price index that occupies the middle position an important liaison. The structural holes largely control the transfer of fluctuations in the price index. When measuring the level of structural holes and calculating the structural holes' hierarchy for each price index in the network structure, a higher hierarchy means that the price index is of more importance in the transfer process. The formulation of price indices *i* in the calculation of the network structure hierarchy is
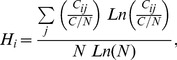
(6)where *N* is the number of vertices in the individual network of vertices (price index) *i*, *C/N* is the mean value of the constraint on each vertex, and the vertices constraint *C_ij_* is the extent to which the vertices in the network have the capacity of using structural holes. The relevant equation can be expressed as
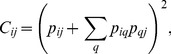
(7)where *p_iq_* is the share of the relationships of vertices *j* compared to all relationships of vertices *i*.

By calculating the structural holes hierarchy of each price index (see Table S3), the structural holes hierarchy of the price indices with a degree of influence of no less than 10 (weighted degree> = 10) are shown in Table 4. Most of the top 10 price indices are PPI-type indices, which mean that, in the PIWN, the PPI has strong transmission effects; however, the price indices of food and print media in the producer price index have stronger transmission effects, with a degree of influence of more than 25. Therefore, to moderate the fluctuation of commodity price indices, it is important to regulate and control these price indices with strong transmission effects. The results of the experiment show that price indices with a structural holes hierarchy of more than 0.02 have a degree of influence of less than 8. These results show that, even though some price indices in the network structure have a small degree of influence, they play important roles in the transmission of price fluctuations. At the same time, we found that price fluctuations between group clusters are conducted by their mutual price indices. As shown in Figure 6, the black nodes are conducting mediums of 2 group clusters.

**Table 4:** Structural holes hierarchy of price indices with a degree of influence no less than 10 (top 10 price indices).

Rank	Type of price index	Weighted degree	Hierarchy
1	CPI(Touring and Outing)	13.05	0.01843
2	PPI(Processing of Food from Agricultural Products)	28.77	0.01412
3	PPI(Food)	32.70	0.01405
4	PPI(Printing, Reproduction of Recording Media)	27.84	0.01378
5	RPI(Furniture)	27.79	0.01312
6	PPI(Food Industry)	30.13	0.01269
7	CPI(Intercity Traffic Fare)	11.43	0.01253
8	CPI(Transportation)	11.38	0.01253
9	API	36.18	0.01242
10	PPI(Raw Materials Industry)	26.43	0.01236

### Transmission distance and path

When one of the price indices changes, how far can the transmission process go, and what is the transmission path? To answer this question, we should know the shortest transmission distance between price indices. The shortest transmission distance in the PIWN can be determined by calculating the shortest path. This distance can be defined as the minimum number of edges through which the two price indices pass in the network structure. When a price index changes, we could measure its longest impact distance by calculating the shortest path between this price index and others. Additionally, we could know the impact distance of the whole price indices system by finding the average shortest path of the PIWN. Thus, when a price index changes, we could know which price index will be affected next.

**Figure 8:**
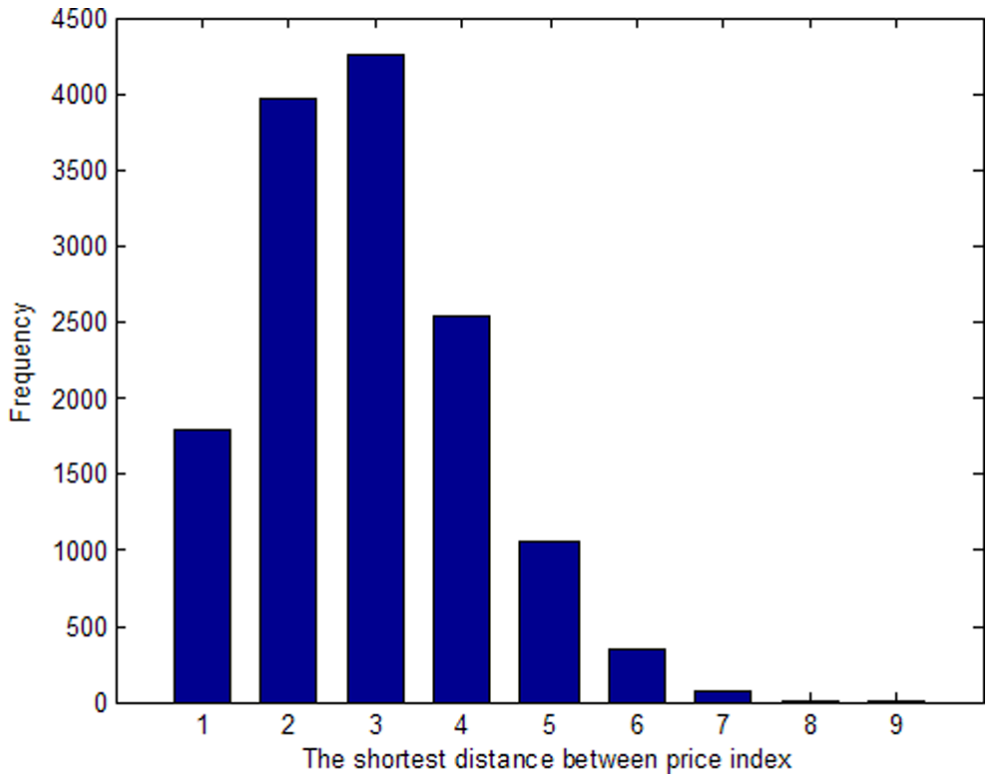
Shortest distance and frequency between price indices. Following a normal distribution with a confidence level of 0.78.

As shown in Figure 8, the transmission distances of the price indices follow a normal distribution. A distance of 1 means that the two price indices in the PIWN are connected directly. Changes in either price index will affect the other. The impact distance is considered to be 1. A distance of 2 means that the two price indices are connected by another price index (connected indirectly). Thus, the impact distance is expanded to 2. After continuing in this fashion, Figure 8 shows that the transmission distances of the price indices are concentrated between 2 and 4. Thus, generally the transmission distances of most of the price indices are 2–4, which means that, when a price index changes, only 2–4 distances are needed to affect the majority. The longest fluctuation transmission distance is 9 in the PIWN, which means that, when a certain price index changes, the whole price index system will be affected within a distance of 9. The average shortest path length is 2.55. The network structure cohesion index based on “distance” is 0.953. So, the transmission between price indices in the network structure is relatively fast, has a small-world effect, and, on average, can be completed by one price index. The results also show that, in real-world inflation, a vertex often has an impact on all of the commodity prices before effective control measures are taken.

**Figure 9:**
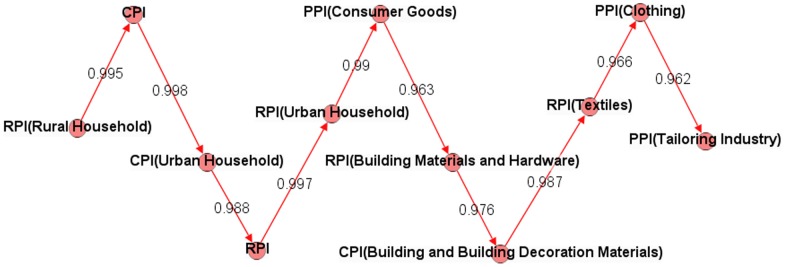
RPI (Rural Household) transmission paths with the strongest correlations.

We analyzed the paths in the PIWN and found the transmission path based on the weights of the edges. Taking RPI (Rural Household) as an example (the value of its weighted degree is the largest), when RPI (Rural Household) changes, the most correlated node is CPI, and the node most correlated with CPI is CPI (Urban Household). Following this process, we could find out other nodes. According to the shortest path that we obtained above, the longest transmission distance is 9; as a result, when we found the 10th node, the RPI (Rural Household) had already affected the whole system. The transmission path of the RPI (Rural Household) is shown in Figure 9, with the correlations between the price indices (weights of the edges) all above 0.96. Of course, we had only considered the path with the strongest correlations. In reality, the nodes on the transmission path will pass the fluctuation on to other nodes. We could use the same method to find all of the transmission paths and then to more fully know the transmission process of the price indices.

## Discussion and Conclusions

This paper analyzed the features in the correlation structure of price indices by the methods that involve complex networks. Theoretically, we considered relationships between many factors and conducted the research with a structured view, while the traditional econometrics can only analyze relationships between a few factors. In practice, this approach could help us to know more about the interactions of price indices by analyzing the features of the correlation structure. This analysis not only shows us the principles of the interactions of price indices in the economic system but also provides us with effective proofs for macroeconomic control policies for the government. Finding out the key price indices could make us aware of which control object is the key target; analyzing the group clusters could show us the influences between price indices, and then, we could suggest to the government how to stabilize the market price by regulating sparsely. Analyzing the transmission medium shows us the medium level of every price index, and the government could prevent the expansion of fluctuations by controlling the mediums. Measuring the transmission distance and discovering the path tells us the impact scale and its transmission path when there are fluctuations in price indices; these transmission paths provide references when making warning policies.

An issue that should be investigated further is how to study the relationships that involve many variables if the number of affecting factors in an economic system continues to increase; there are hundreds or even thousands of factors that interact with each other. As shown in the literature review, most of the studies on the relationship between variables use theories and methods of econometrics such as linear regressions, causality tests, and co-integration tests. However, the purpose of these econometric models is not to include all of the variables; instead, the purpose is to include only the most significant factors. If too many variables are introduced, then the model will be too complex and the research will become meaningless. The number of objects included in traditional econometric methods is two; even if there are multiple variables, the analysis is performed with only two variables. Additionally, many of the objects included are macroscopic. Thus, it is easy to ignore micro-level variables. These variables have correlations and influence, and they interact with each other to form a complicated relationship system. Macroscopic changes are caused by changes in the complexity of these variables. Existing research rarely addresses these issues. Complex network theory provides a good foundation to address the problem of complexity. Relationships between variables are abstracted into network vertices and edges, applying research on the relationship between numerous variables in the network. The complex network analysis method provides a range of parameters and can be combined with economic theories and analytical methods to study complex issues in the price indices system and the financial system and even in the economic system.

By building the price index correlation network structure model, the method not only can be used to study the correlation structure of price indices but also can be applied to other fields, such as the analysis of the correlation between various elements of commodity markets, the analysis of stock price linkages in the financial markets, research on input-output relationships in industrial chains, and research on correlation relationships between various national economic indices. These issues involve more variables and more complex relationships. Further research should aim to define complex relationships between more variables and to combine qualitative and quantitative analysis methods.

## Supporting Information

Table S1 **The value of the weighted degree of price index (PDF).**
(PDF)

Table S2 **The class of group clusters (PDF).**
(PDF)

Table S3 **Structural holes hierarchy of price indices (PDF).**
(PDF)
