# Supplementary material for: Features of the Correlation Structure of Price Indices
Source: PLoS One. 2013 Apr 8;8(4):e61091. doi: 10.1371/journal.pone.0061091 (PMC3620382; doi:10.1371/journal.pone.0061091)
Supplement: Table S1 — The value of the weighted degree of price index (PDF). (PDF) [file pone.0061091.s001.pdf]

Table S1. The value of the weighted degree of price index

Rank	Type of price index	Weighted Degree
1	RPI(Rural Household)	42.18656
2	PPI(Articles for Daily Use)	41.69579
3	RPI	40.59030
4	PPI(Manufacture of General Purpose Machinery)	39.92459
5	RPI(Urban Household)	39.64424
6	CPI(Rural Household)	39.13687
7	PPI(Manufacture of Special Purpose Machinery)	39.00185
8	PPI(Manufacture of Artwork and Other Manufacturing)	38.63017
9	RPI(Building Materials and Hardware)	38.58309
10	PPI(Consumer Goods)	37.62457
11	CPI	36.26456
12	API	36.17860
13	PPI(Paper Industry)	36.11174
14	PPI(Manufacture of Paper and Paper Products)	35.29338
15	API(Fishery Products)	34.87342
16	CPI(Urban Household)	34.61388
17	CPI(Building and Building Decoration Materials)	34.51912
18	PPI(Processing Industry)	33.17526
19	PPI(Food)	32.70492
20	API(Semi-mechanized Farm Tools)	32.70441
21	RPI(Textiles)	32.63454
22	CPI(Dining Out)	31.68390
23	RFPPI(Building Materials)	31.41370
24	RFPPI(Timber and Paper Pulp)	31.07498
25	PPI(Manufacture of Metal Products)	30.97866
26	PPI(Manufacture of Beverages)	30.38252
27	PPI	30.16932
28	PPI(Food Industry)	30.13110
29	PPI(Processing of Foodstuff)	29.52091
30	PPI(Clothing)	29.32681
31	RPI(Food)	29.27867
32	API(Farm Handtools)	29.00980
33	PPI(Building Materials Industry)	28.85871
34	PPI(Processing of Food from Agricultural Products)	28.77093
35	PPI(Means of Production)	28.51938
36	CPI(Food)	28.41160
37	PPI(Manufacture of Non-metallic Mineral Products)	28.23350
38	PPI(Printing, Reproduction of Recording Media)	27.84289
39	RPI(Furniture)	27.78815
40	PPI(Timber Industry)	27.74870
41	RFPPI	27.62937

Rank	Type of price index	Weighted Degree
42	CPI(Daily Use Household Articles)	27.17604
43	CPI(Clothing Material)	27.06978
44	CPI(Residence)	26.83278
45	API(Mechanized Farm Machinery)	26.59506
46	PPI(Manufacture of Transport Equipment)	26.47834
47	PPI(Manufacture of Raw Chemical Materials and Chemical Products)	26.45613
48	PPI(Raw Materials Industry)	26.43406
49	PPI(Processing of Timber, Manufacture of Wood, Bamboo,)	25.96986
50	PPI(Manufacture of Communication Equipment, Computers and)	25.96191
51	PPI(Chemical Industry)	25.56021
52	PPI(Manufacture of Measuring Instruments and Machinery for)	24.74957
53	API(Freshwater Fish)	24.60081
54	PPI(Tailoring Industry)	23.87075
55	RPI(Articles for Daily Use)	23.73754
56	CPI(Personal Services)	23.69675
57	RFPPI(Raw Chemical Materials)	23.46138
58	PPI(Machine Manufac- turing Industry)	22.92237
59	RPI(Meat, Poultry and Processed Products)	22.78490
60	CPI(Meat, Poultry and Processed Products)	22.74652
61	CPI(Household Services and Maintenance and Renovation)	22.73583
62	API(Cattle and Buffaloes (gross weight))	22.71147
63	PPI(Durable Consumer Goods)	22.65671
64	RPI(Beverages, Tobacco and Liquor)	22.39143
65	CPI(Health Care Appliances and Articles)	22.13610
66	PPI(Metallurgical Industry)	21.81263
67	PPI(Mining & Quarrying Industry)	21.79330
68	API(Pig (gross weight))	21.70854
69	API(Animal Husbandry Products)	21.18750
70	API(Service for Agricultural Production)	21.06638
71	API(Pesticide and Its Appliances)	20.73162
72	API(Oil for Farm Machinery)	20.70173
73	API(Chemical Fertilizer)	20.57037
74	PPI(Mining and Processing of Nonmetal Ores)	20.22700
75	RFPPI(Fuel and Power)	20.11008
76	CPI(Household Facilities, Articles and Services)	20.04999
77	CPI(Transportation Facility)	19.95963
78	CPI(Water, Electricity and Fuels)	19.93886
79	RPI(Fuels)	19.45278
80	API(Commodity Animals)	19.27352
81	RFPPI(Textile Materials)	19.23547
82	API(Sheep and Goats (gross weight))	19.10939
83	CPI(Clothing Manufacturing Services)	19.09660
84	CPI(Oil or Fat)	19.02774

Rank	Type of price index	Weighted Degree
85	PPI(Mining and Processing of Ferrous Metal Ores)	18.80463
86	RPI(Aquatic Products)	18.57876
87	PPI(Smelting and Pressing of Ferrous Metals)	18.53838
88	CPI(Bed Articles)	18.44161
89	PPI(Extraction of Petroleum and Natural Gas)	18.30723
90	RPI(Oil or Fat)	18.18970
91	CPI(Fuels and Parts)	18.09249
92	CPI(Western Medicine)	18.05539
93	PPI(Manufacture of Textile Wearing Apparel, Footware, and Caps)	17.80424
94	API(Forage)	17.39962
95	RFPPI(Ferrous Metals)	17.21255
96	API(Milk)	17.19318
97	PPI(Recycling and Disposal of Waste)	17.01570
98	CPI(Tobacco, Liquor and Articles)	16.88201
99	PPI(Coal Industry)	16.82283
100	CPI(Liquor)	16.17631
101	CPI(Aquatic Products)	15.93107
102	RPI(Traditional Chinese and Western Medicines and Health Care Articles)	15.68090
103	PPI(Manufacture of Plastics)	15.67946
104	PPI(Petroleum Industry)	15.57548
105	CPI(Sanitation Articles)	15.30972
106	PPI(Cultural, Educational & Handicrafts Articles)	15.00993
107	PPI(Manufacture of Furniture)	14.94723
108	CPI(Personal Articles and Services)	14.62109
109	RPI(Cosmetics)	14.54520
110	PPI(Leather Industry)	13.91824
111	PPI(Processing of Petroleum, Coking, Processing of Nuclear Fuel)	13.86631
112	CPI(Interior Decorations)	13.74079
113	CPI(Durable Consumer Goods)	13.28997
114	CPI(Touring and Outing)	13.04715
115	API(Poultry (gross weight))	12.54152
116	API(Eggs)	12.21440
117	API(Oil-bearing Crops)	11.46163
118	CPI(Intercity Traffic Fare)	11.43466
119	CPI(Transportation)	11.37632
120	RPI(Household Appliances, Music and Video Equipment)	11.18481
121	PPI(Manufacture of Articles for Culture, Education and Sport Activities)	11.15512
122	CPI(Transportation and Communication)	10.81337
123	RPI(Transportation and Communication Appliances)	10.70712
124	PPI(Manufacture of Leather, Fur, Feather and Related Products)	10.53261
125	CPI(Private Housing)	10.33800
126	RPI(Cultural and Office Appliances)	9.65284
127	PPI(Manufacture of Chemical Fibers)	9.39575

Rank	Type of price index	Weighted Degree
128	PPI(Mining and Washing of Coal)	8.96611
129	RFPPI(Agricultural Products)	8.90817
130	PPI(Manufacture of Rubber)	8.89425
131	PPI(Manufacture of Electrical Machinery and Equipment)	8.83531
132	API(Forestry Products)	8.82198
133	PPI(Production and Supply of Gas)	8.82125
134	CPI(Cosmetics)	7.85374
135	API(Corn)	7.62977
136	CPI(Durable Consumer Goods for Cultural and)	7.15431
137	CPI(Personal Ornaments)	7.07394
138	API(Seawater Fish)	6.77591
139	CPI(Clothing)	6.36783
140	API(Beans)	6.28799
141	RPI(Gold, Silver and Jewelry)	6.15286
142	API(Planting Products)	6.14069
143	PPI(Mining and Processing of Non-Ferrous Metal Ores)	5.51784
144	PPI(Manufacture of Textile)	5.42247
145	PPI(Textile Industry)	5.37625
146	CPI(Footgear and Hats)	5.34408
147	CPI(Health Care and Personal Articles)	5.27405
148	API(Cereal)	4.83710
149	CPI(Grain)	4.81780
150	RPI(Grain)	4.81750
151	API(Wheat)	4.75914
152	RPI(Garments, Shoes and Hats)	4.62850
153	CPI(Renting)	4.60025
154	CPI(Garments)	4.46726
155	CPI(Eggs)	4.46246
156	RPI(Eggs)	4.46246
157	API(Other Means of Agricultural Production)	4.21380
158	API(Rice)	3.93023
159	RFPPI(Nonferrous Metals)	3.84207
160	PPI(Smelting and Pressing of Non-ferrous Metals)	3.79135
161	CPI(Health Care)	3.60792
162	CPI(Fresh Vegetables)	2.83614
163	CPI(Cultural Articles)	2.57567
164	CPI(Vegetables)	1.99311
165	RPI(Vegetables)	1.98744
166	CPI(Dried and Fresh Melons and Fruits)	1.97478
167	CPI(Tuition and Child Care)	1.96543
168	CPI(Education)	1.95735
169	RPI(Dried and Fresh Melons and Fruits)	1.95711
170	CPI(Newspapers and Magazines)	1.95212

Rank	Type of price index	Weighted Degree
171	CPI(Cultural and Recreational Articles)	1.94161
172	CPI(Fresh Fruits)	1.94016
173	RPI(Books, Newspapers, Magazines and Electronic Publications)	1.93036
174	CPI(Recreation, Education and Culture Articles)	1.92527
175	CPI(Incity Traffic Fare)	1.78856
176	CPI(Traditional Chinese Medicine)	1.78117
177	CPI(Tobacco)	1.75585
178	PPI(Power Industry)	0.99958
179	PPI(Production and Supply of Electric Power and Heat Power)	0.99958
180	#N/A	0.96298
181	CPI(Expenditure on Culture and Recreation)	0.96298
182	CPI(Communication)	0.92233
183	CPI(Communication Service)	0.92233
184	CPI(Communication Facility)	0.89772
185	PPI(Manufacture of Tobacco)	0.89772
186	API(Sugar)	0.89664
187	PPI(Manufacture of Medicines)	0.85612
188	API(Cotton)	0.85176
189	CPI(Medical Instrument and Articles)	0.82808
190	CPI(Teaching Materials and Reference Books)	0.82808
191	CPI(Fees for Vehicles Use and Maintenance)	0.82501
