# Supplementary material for: Features of the Correlation Structure of Price Indices
Source: PLoS One. 2013 Apr 8;8(4):e61091. doi: 10.1371/journal.pone.0061091 (PMC3620382; doi:10.1371/journal.pone.0061091)
Supplement: Table S2 — The class of group clusters (PDF). (PDF) [file pone.0061091.s002.pdf]

Table S2. The class of group clusters

Type of price index	*k*-cores class
RPI(Sports and Recreation Articles)	0
API(Vegetable)	0
API(Fruit)	0
PPI(Production and Supply of Water)	0
CPI(Medical Instrument and Articles)	1
CPI(Health Care)	1
CPI(Fees for Vehicles Use and Maintenance)	1
CPI(Incity Traffic Fare)	1
CPI(Communication)	1
CPI(Communication Facility)	1
CPI(Communication Service)	1
CPI(Teaching Materials and Reference Books)	1
CPI(Expenditure on Culture and Recreation)	1
API(Cotton)	1
API(Sugar)	1
PPI(Power Industry)	1
PPI(Manufacture of Tobacco)	1
PPI(Manufacture of Medicines)	1
PPI(Production and Supply of Electric Power and Heat Power)	1
CPI(Vegetables)	2
CPI(Fresh Vegetables)	2
CPI(Dried and Fresh Melons and Fruits)	2
CPI(Fresh Fruits)	2
CPI(Tobacco)	2
CPI(Traditional Chinese Medicine)	2
CPI(Recreation, Education and Culture Articles)	2
CPI(Education)	2
CPI(Tuition and Child Care)	2
CPI(Cultural and Recreational Articles)	2
CPI(Newspapers and Magazines)	2
RPI(Vegetables)	2
RPI(Dried and Fresh Melons and Fruits)	2
RPI(Books, Newspapers, Magazines and Electronic Publications)	2
CPI(Health Care Services)	3
CPI(Cultural Articles)	3
CPI(Grain)	4
CPI(Eggs)	4
CPI(Health Care and Personal Articles)	4
RPI(Grain)	4
RPI(Eggs)	4
API(Planting Products)	4

Type of price index	k-cores class
API(Cereal)	4
API(Wheat)	4
API(Rice)	4
PPI(Mining and Processing of Non-Ferrous Metal Ores)	4
PPI(Smelting and Pressing of Non-ferrous Metals)	4
RFPPI(Nonferrous Metals)	4
CPI(Clothing)	5
CPI(Garments)	5
CPI(Footgear and Hats)	5
CPI(Durable Consumer Goods for Cultural and)	5
CPI(Renting)	5
RPI(Garments, Shoes and Hats)	5
API(Other Means of Agricultural Production)	5
PPI(Manufacture of Electrical Machinery and Equipment)	5
RPI(Cultural and Office Appliances)	6
API(Corn)	6
API(Beans)	6
PPI(Textile Industry)	6
PPI(Manufacture of Textile)	6
RFPPI(Agricultural Products)	6
CPI(Personal Ornaments)	7
RPI(Gold, Silver and Jewelry)	7
API(Seawater Fish)	7
CPI(Cosmetics)	8
CPI(Transportation and Communication)	8
CPI(Transportation)	8
CPI(Intercity Traffic Fare)	8
CPI(Touring and Outing)	8
RPI(Household Appliances, Music and Video Equipment)	8
API(Forestry Products)	8
PPI(Manufacture of Rubber)	8
PPI(Production and Supply of Gas)	8
CPI(Private Housing)	9
PPI(Manufacture of Leather, Fur, Feather and Related Products)	9
PPI(Manufacture of Chemical Fibers)	9
PPI(Mining and Washing of Coal)	10
RPI(Transportation and Communication Appliances)	11
API(Oil-bearing Crops)	11
CPI(Bed Articles)	12
API(Poultry (gross weight))	12
API(Eggs)	12
PPI(Leather Industry)	12
PPI(Manufacture of Articles for Culture, Education and Sport Activities)	12

Type of price index	k-cores class
CPI(Durable Consumer Goods)	13
CPI(Interior Decorations)	14
CPI(Western Medicine)	14
CPI(Sanitation Articles)	14
CPI(Fuels and Parts)	14
RPI(Cosmetics)	14
RPI(Traditional Chinese and Western Medicines and Health Care Articles)	14
API(Oil for Farm Machinery)	14
PPI(Petroleum Industry)	14
PPI(Machine Manufac- turing Industry)	14
PPI(Extraction of Petroleum and Natural Gas)	14
PPI(Manufacture of Furniture)	14
PPI(Processing of Petroleum, Coking, Processing of Nuclear Fuel)	14
PPI(Recycling and Disposal of Waste)	14
CPI(Tobacco, Liquor and Articles)	15
CPI(Liquor)	15
CPI(Clothing Material)	15
CPI(Clothing Manufacturing Services)	15
CPI(Household Facilities, Articles and Services)	15
CPI(Daily Use Household Articles)	15
CPI(Household Services and Maintenance and Renovation)	15
CPI(Health Care Appliances and Articles)	15
CPI(Personal Articles and Services)	15
CPI(Personal Services)	15
CPI(Transportation Facility)	15
CPI(Residence)	15
CPI(Water, Electricity and Fuels)	15
RPI(Beverages, Tobacco and Liquor)	15
RPI(Articles for Daily Use)	15
RPI(Furniture)	15
RPI(Fuels)	15
API(Service for Agricultural Production)	15
PPI	15
PPI(Metallurgical Industry)	15
PPI(Coal Industry)	15
PPI(Chemical Industry)	15
PPI(Cultural, Educational & Handicrafts Articles)	15
PPI(Mining and Processing of Ferrous Metal Ores)	15
PPI(Mining and Processing of Nonmetal Ores)	15
PPI(Printing, Reproduction of Recording Media)	15
PPI(Manufacture of Raw Chemical Materials and Chemical Products)	15
PPI(Manufacture of Plastics)	15
PPI(Smelting and Pressing of Ferrous Metals)	15

Type of price index	k-cores class
PPI(Manufacture of Transport Equipment)	15
PPI(Manufacture of Communication Equipment, Computers and)	15
PPI(Manufacture of Measuring Instruments and Machinery for)	15
PPI(Means of Production)	15
PPI(Mining & Quarrying Industry)	15
PPI(Raw Materials Industry)	15
PPI(Durable Consumer Goods)	15
RFPPI	15
RFPPI(Fuel and Power)	15
RFPPI(Ferrous Metals)	15
RFPPI(Raw Chemical Materials)	15
RFPPI(Timber and Paper Pulp)	15
RFPPI(Textile Materials)	15
CPI(Oil or Fat)	16
CPI(Aquatic Products)	16
RPI(Oil or Fat)	16
RPI(Aquatic Products)	16
API(Farm Handtools)	16
API(Forage)	16
API(Mechanized Farm Machinery)	16
API(Chemical Fertilizer)	16
API(Pesticide and Its Appliances)	16
PPI(Manufacture of Beverages)	16
PPI(Manufacture of Textile Wearing Apparel, Footware, and Caps)	16
PPI(Manufacture of Metal Products)	16
PPI(Processing Industry)	16
CPI	17
CPI(Urban Household)	17
CPI(Rural Household)	17
API	17
RPI	17
RPI(Urban Household)	17
RPI(Rural Household)	17
CPI(Food)	17
CPI(Meat, Poultry and Processed Products)	17
CPI(Dining Out)	17
CPI(Building and Building Decoration Materials)	17
RPI(Food)	17
RPI(Meat, Poultry and Processed Products)	17
RPI(Textiles)	17
RPI(Building Materials and Hardware)	17
API(Commodity Animals)	17
API(Semi-mechanized Farm Tools)	17

**Type of price index**	**k-cores class**
API(Animal Husbandry Products)	17
API(Pig (gross weight))	17
API(Cattle and Buffaloes (gross weight))	17
API(Sheep and Goats (gross weight))	17
API(Milk)	17
API(Fishery Products)	17
API(Freshwater Fish)	17
PPI(Building Materials Industry)	17
PPI(Timber Industry)	17
PPI(Food Industry)	17
PPI(Tailoring Industry)	17
PPI(Paper Industry)	17
PPI(Processing of Food from Agricultural Products)	17
PPI(Processing of Foodstuff)	17
PPI(Processing of Timber, Manufacture of Wood, Bamboo,)	17
PPI(Manufacture of Paper and Paper Products)	17
PPI(Manufacture of Non-metallic Mineral Products)	17
PPI(Manufacture of General Purpose Machinery)	17
PPI(Manufacture of Special Purpose Machinery)	17
PPI(Manufacture of Artwork and Other Manufacturing)	17
PPI(Consumer Goods)	17
PPI(Food)	17
PPI(Clothing)	17
PPI(Articles for Daily Use)	17
RFPPI(Building Materials)	17
