# Supplementary material for: Features of the Correlation Structure of Price Indices
Source: PLoS One. 2013 Apr 8;8(4):e61091. doi: 10.1371/journal.pone.0061091 (PMC3620382; doi:10.1371/journal.pone.0061091)
Supplement: Table S3 — Structural holes hierarchy of price indices (PDF). (PDF) [file pone.0061091.s003.pdf]

Table S3. Structural holes hierarchy of price indices

Rank	Type of price index	Hierarchy
1	CPI(Medical Instrument and Articles)	1.00000
2	CPI(Health Care Services)	1.00000
3	CPI(Fees for Vehicles Use and Maintenance)	1.00000
4	CPI(Communication)	1.00000
5	CPI(Communication Facility)	1.00000
6	CPI(Communication Service)	1.00000
7	CPI(Teaching Materials and Reference Books)	1.00000
8	CPI(Expenditure on Culture and Recreation)	1.00000
9	API(Cotton)	1.00000
10	API(Sugar)	1.00000
11	PPI(Power Industry)	1.00000
12	PPI(Manufacture of Tobacco)	1.00000
13	PPI(Manufacture of Medicines)	1.00000
14	PPI(Production and Supply of Electric Power and Heat Power)	1.00000
15	CPI(Fresh Vegetables)	0.05220
16	CPI(Eggs)	0.03159
17	RPI(Eggs)	0.03159
18	CPI(Health Care)	0.03113
19	API(Planting Products)	0.02852
20	CPI(Durable Consumer Goods for Cultural and)	0.02581
21	PPI(Mining and Processing of Non-Ferrous Metal Ores)	0.02121
22	CPI(Touring and Outing)	0.01843
23	CPI(Footgear and Hats)	0.01822
24	CPI(Health Care and Personal Articles)	0.01792
25	PPI(Processing of Food from Agricultural Products)	0.01412
26	PPI(Food)	0.01405
27	PPI(Printing, Reproduction of Recording Media)	0.01378
28	RFPPI(Agricultural Products)	0.01376
29	PPI(Manufacture of Electrical Machinery and Equipment)	0.01328
30	RPI(Furniture)	0.01312
31	PPI(Food Industry)	0.01269
32	CPI(Transportation)	0.01253
33	CPI(Intercity Traffic Fare)	0.01253
34	API	0.01242
35	PPI(Raw Materials Industry)	0.01236
36	PPI(Means of Production)	0.01226
37	PPI(Manufacture of Transport Equipment)	0.01224
38	API(Forestry Products)	0.01224
39	PPI	0.01222
40	PPI(Durable Consumer Goods)	0.01211
41	CPI(Private Housing)	0.01190

Rank	Type of price index	Hierarchy
42	CPI	0.01189
43	CPI(Food)	0.01185
44	RPI(Food)	0.01184
45	RPI(Household Appliances, Music and Video Equipment)	0.01174
46	PPI(Manufacture of Measuring Instruments and Machinery for)	0.01158
47	CPI(Daily Use Household Articles)	0.01141
48	PPI(Production and Supply of Gas)	0.01136
49	CPI(Clothing Material)	0.01134
50	RFPPI(Raw Chemical Materials)	0.01118
51	PPI(Timber Industry)	0.01086
52	CPI(Urban Household)	0.01082
53	CPI(Residence)	0.01079
54	RFPPI(Timber and Paper Pulp)	0.01072
55	RPI(Rural Household)	0.01063
56	PPI(Processing of Timber, Manufacture of Wood, Bamboo,)	0.01031
57	RPI(Garments, Shoes and Hats)	0.01031
58	PPI(Processing Industry)	0.01016
59	CPI(Building and Building Decoration Materials)	0.01014
60	PPI(Manufacture of Paper and Paper Products)	0.01004
61	PPI(Articles for Daily Use)	0.01002
62	RPI(Cultural and Office Appliances)	0.00999
63	PPI(Manufacture of Rubber)	0.00998
64	RPI(Meat, Poultry and Processed Products)	0.00994
65	CPI(Vegetables)	0.00994
66	RPI(Vegetables)	0.00994
67	RPI	0.00993
68	RPI(Building Materials and Hardware)	0.00975
69	PPI(Paper Industry)	0.00971
70	PPI(Building Materials Industry)	0.00969
71	API(Chemical Fertilizer)	0.00953
72	PPI(Manufacture of Furniture)	0.00951
73	CPI(Rural Household)	0.00947
74	RFPPI	0.00947
75	PPI(Manufacture of General Purpose Machinery)	0.00944
76	CPI(Meat, Poultry and Processed Products)	0.00941
77	PPI(Manufacture of Communication Equipment, Computers and)	0.00940
78	CPI(Oil or Fat)	0.00933
79	CPI(Sanitation Articles)	0.00910
80	API(Pesticide and Its Appliances)	0.00906
81	API(Fishery Products)	0.00901
82	RFPPI(Building Materials)	0.00900
83	CPI(Transportation Facility)	0.00893
84	PPI(Consumer Goods)	0.00891

Rank	Type of price index	Hierarchy
85	PPI(Manufacture of Non-metallic Mineral Products)	0.00889
86	PPI(Machine Manufac- turing Industry)	0.00884
87	API(Beans)	0.00878
88	PPI(Chemical Industry)	0.00872
89	PPI(Manufacture of Special Purpose Machinery)	0.00870
90	PPI(Processing of Foodstuff)	0.00856
91	PPI(Extraction of Petroleum and Natural Gas)	0.00835
92	RPI(Urban Household)	0.00832
93	RPI(Articles for Daily Use)	0.00822
94	CPI(Bed Articles)	0.00818
95	CPI(Clothing)	0.00811
96	PPI(Manufacture of Raw Chemical Materials and Chemical Products)	0.00796
97	PPI(Clothing)	0.00787
98	PPI(Mining and Processing of Ferrous Metal Ores)	0.00787
99	CPI(Personal Services)	0.00782
100	PPI(Manufacture of Metal Products)	0.00775
101	CPI(Fuels and Parts)	0.00767
102	RPI(Oil or Fat)	0.00763
103	PPI(Coal Industry)	0.00756
104	PPI(Manufacture of Chemical Fibers)	0.00750
105	PPI(Petroleum Industry)	0.00744
106	API(Pig (gross weight))	0.00740
107	API(Mechanized Farm Machinery)	0.00737
108	CPI(Garments)	0.00727
109	CPI(Household Services and Maintenance and Renovation)	0.00720
110	RPI(Textiles)	0.00717
111	CPI(Clothing Manufacturing Services)	0.00713
112	PPI(Manufacture of Artwork and Other Manufacturing)	0.00709
113	PPI(Smelting and Pressing of Ferrous Metals)	0.00707
114	PPI(Recycling and Disposal of Waste)	0.00685
115	RFPPI(Textile Materials)	0.00683
116	PPI(Mining and Processing of Nonmetal Ores)	0.00676
117	RFPPI(Ferrous Metals)	0.00673
118	PPI(Mining & Quarrying Industry)	0.00661
119	CPI(Water, Electricity and Fuels)	0.00653
120	API(Animal Husbandry Products)	0.00641
121	API(Oil-bearing Crops)	0.00635
122	CPI(Personal Ornaments)	0.00633
123	CPI(Health Care Appliances and Articles)	0.00631
124	PPI(Metallurgical Industry)	0.00627
125	CPI(Cosmetics)	0.00620
126	RPI(Gold, Silver and Jewelry)	0.00620
127	API(Service for Agricultural Production)	0.00608

Rank	Type of price index	Hierarchy
128	PPI(Manufacture of Articles for Culture, Education and Sport Activities)	0.00605
129	API(Semi-mechanized Farm Tools)	0.00596
130	PPI(Manufacture of Beverages)	0.00593
131	API(Oil for Farm Machinery)	0.00593
132	RFPPI(Fuel and Power)	0.00586
133	CPI(Household Facilities, Articles and Services)	0.00574
134	RPI(Beverages, Tobacco and Liquor)	0.00570
135	API(Farm Handtools)	0.00562
136	PPI(Manufacture of Plastics)	0.00554
137	PPI(Cultural, Educational & Handicrafts Articles)	0.00544
138	PPI(Tailoring Industry)	0.00528
139	CPI(Durable Consumer Goods)	0.00522
140	API(Corn)	0.00520
141	API(Sheep and Goats (gross weight))	0.00517
142	CPI(Transportation and Communication)	0.00514
143	API(Poultry (gross weight))	0.00497
144	API(Eggs)	0.00497
145	CPI(Western Medicine)	0.00470
146	API(Forage)	0.00433
147	RPI(Aquatic Products)	0.00431
148	API(Commodity Animals)	0.00426
149	CPI(Liquor)	0.00412
150	API(Freshwater Fish)	0.00404
151	API(Cattle and Buffaloes (gross weight))	0.00403
152	CPI(Tobacco, Liquor and Articles)	0.00398
153	RPI(Fuels)	0.00380
154	PPI(Leather Industry)	0.00373
155	CPI(Grain)	0.00372
156	RPI(Grain)	0.00372
157	API(Cereal)	0.00372
158	API(Wheat)	0.00372
159	CPI(Aquatic Products)	0.00371
160	RPI(Traditional Chinese and Western Medicines and Health Care Articles)	0.00365
161	API(Milk)	0.00357
162	CPI(Traditional Chinese Medicine)	0.00343
163	CPI(Renting)	0.00310
164	PPI(Manufacture of Leather, Fur, Feather and Related Products)	0.00306
165	API(Other Means of Agricultural Production)	0.00306
166	PPI(Textile Industry)	0.00300
167	PPI(Manufacture of Textile)	0.00300
168	CPI(Dining Out)	0.00299
169	PPI(Processing of Petroleum, Coking, Processing of Nuclear Fuel)	0.00279
170	RPI(Cosmetics)	0.00277

Rank	Type of price index	Hierarchy
171	CPI(Personal Articles and Services)	0.00276
172	PPI(Manufacture of Textile Wearing Apparel, Footware, and Caps)	0.00243
173	RFPPI(Nonferrous Metals)	0.00211
174	PPI(Smelting and Pressing of Non-ferrous Metals)	0.00211
175	RPI(Transportation and Communication Appliances)	0.00202
176	CPI(Interior Decorations)	0.00109
177	API(Seawater Fish)	0.00019
178	CPI(Cultural Articles)	0.00014
179	PPI(Mining and Washing of Coal)	0.00009
180	CPI(Dried and Fresh Melons and Fruits)	0.00000
181	CPI(Fresh Fruits)	0.00000
182	CPI(Tobacco)	0.00000
183	CPI(Incity Traffic Fare)	0.00000
184	CPI(Recreation, Education and Culture Articles)	0.00000
185	CPI(Education)	0.00000
186	CPI(Tuition and Child Care)	0.00000
187	CPI(Cultural and Recreational Articles)	0.00000
188	CPI(Newspapers and Magazines)	0.00000
189	RPI(Dried and Fresh Melons and Fruits)	0.00000
190	RPI(Books, Newspapers, Magazines and Electronic Publications)	0.00000
191	API(Rice)	0.00000
192	RPI(Sports and Recreation Articles)	
193	API(Vegetable)	
194	API(Fruit)	
195	PPI(Production and Supply of Water)	
